# Supplementary material for: Cultured Human Foreskin as a Model System for Evaluating Ionizing Radiation-Induced Skin Injury
Source: Int J Mol Sci. 2022 Aug 29;23(17):9830. doi: 10.3390/ijms23179830 (PMC9456202; doi:10.3390/ijms23179830)
Supplement: Supplementary file 1 [file ijms-23-09830-s001.zip › ijms-1854459-supplementary-Figure S2.pdf]

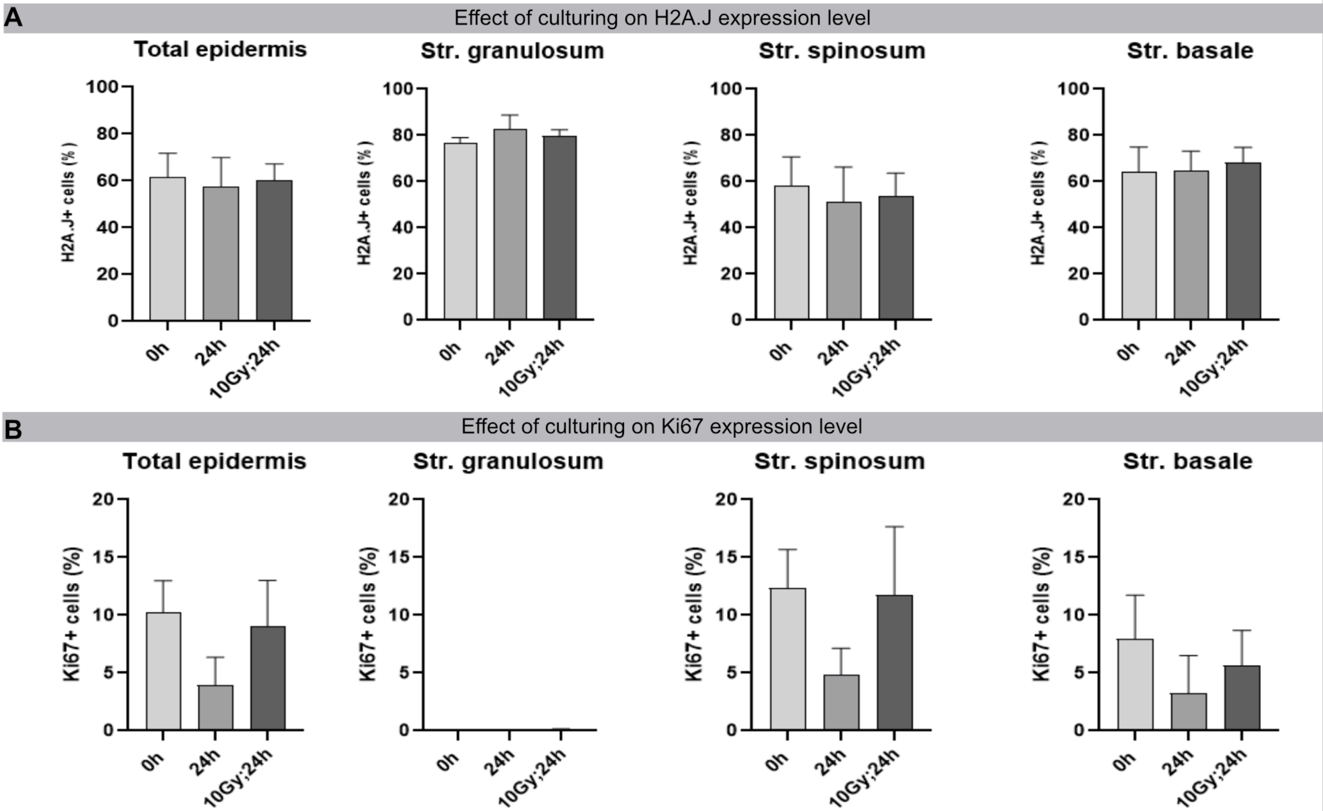

Supplementary Figure S2: Effects of foreskin explant culturing on H2A.J and Ki67 expression: Graphic presentation of the quantification of H2A.J+ (A) and Ki67+ keratinocytes (B) in epidermis of foreskin explants prior to (0h) and 0.5 and 24 hours (0.5h; 24h) after culturing, as well as 24h after IR exposure (10Gy; 24h post-IR). Data are presented as mean ±SE (n= 12).
