# Supplementary figures and images for: Cultured Human Foreskin as a Model System for Evaluating Ionizing Radiation-Induced Skin Injury
Source: Int J Mol Sci. 2022 Aug 29;23(17):9830. doi: 10.3390/ijms23179830 (PMC9456202; doi:10.3390/ijms23179830)

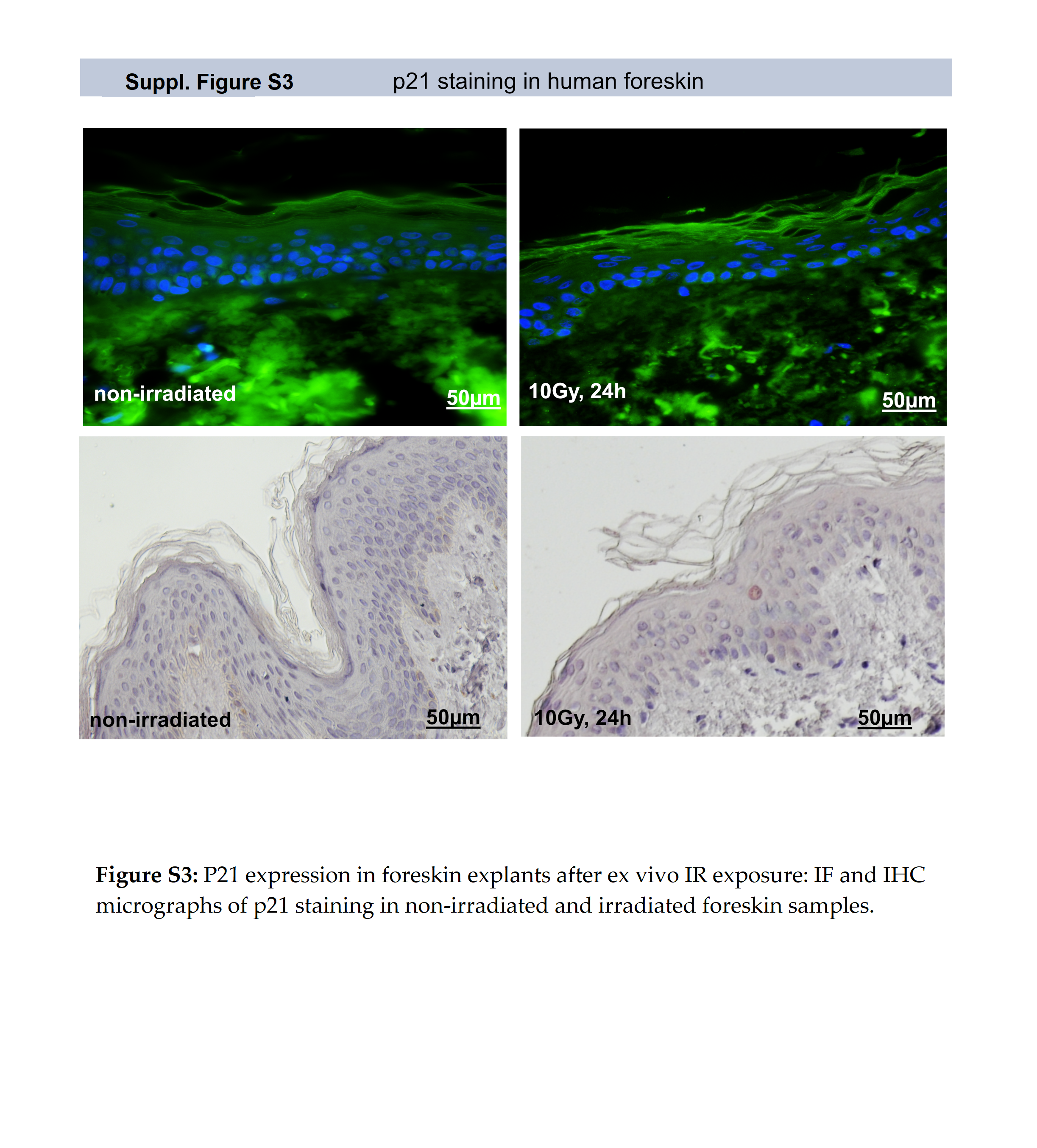

Supplement: Supplementary file 1 [file ijms-23-09830-s001.zip › ijms-1854459-supplementary-Figure S3.tiff]
